# Efficacy of Rituximab in Treatment-Resistant Focal Segmental Glomerulosclerosis With Elevated Soluble Urokinase-Type Plasminogen Activator Receptor and Activation of Podocyte β3 Integrin

**DOI:** 10.1016/j.ekir.2021.10.017

**Published:** 2021-10-30

**Authors:** Michelle A. Hladunewich, Dan Cattran, Sanjeev M. Sethi, Salim S. Hayek, Jing Li, Changli Wei, Sarah I. Mullin, Heather N. Reich, Jochen Reiser, Fernando C. Fervenza

**Affiliations:** 1 Division of Nephrology, Department of Medicine, Sunnybrook Health Sciences Centre, University of Toronto, Toronto, Ontario, Canada; 2 Division of Nephrology, Department of Medicine, University Health Network, University of Toronto, Toronto, Ontario, Canada; 3 Department of Pathology, Mayo Clinic, Rochester, Minnesota, USA; 4 Division of Cardiology, Department of Medicine, University of Michigan, Ann Arbor, Minnesota, USA; 5 Division of Nephrology, Department of Medicine, Rush University Medical Center, Chicago, Illinois, USA; 6 Division of Nephrology, Department of Medicine, Mayo Clinic, Rochester, Minnesota, USA

**Keywords:** focal segmental glomerulosclerosis (FSGS), nephrotic syndrome, rituximab, soluble urokinase-type plasminogen activator receptor (suPAR), treatment resistance

## Abstract

**Introduction:**

Severe, nonresponsive, primary focal segmental glomerular sclerosis (FSGS) can progress to end-stage kidney disease (ESKD) in <5 years. Soluble urokinase-type plasminogen activator receptor (suPAR) may contribute to podocyte effacement by activating podocyte β3 integrin. It has been reported as a potential permeability factor and biomarker for primary FSGS. Rituximab was found to have efficacy in case reports and small series. Whether rituximab is efficacious in patients with treatment-resistant FSGS in the context of high suPAR levels and evidence of podocyte B3 integrin activation remains unknown.

**Methods:**

In this nonblinded, open-label pilot study, the safety and efficacy of rituximab were evaluated in treatment-resistant adult patients with primary FSGS and a suPAR level > 3500 pg/ml with evidence of β3 integrin activation. Rituximab (1 g) was given on days 1 and 15. The primary outcome was proteinuria at 12 months.

**Results:**

Only 13 of 38 screened patients qualified for the study, of whom 9 consented to participate. The baseline proteinuria and glomerular filtration rate (GFR) levels were 7.70 ± 4.61 g/d and 67 ± 38 ml/min, respectively. A transient response at 6 months was noted in 2 patients without a parallel change in suPAR level. At 12 months, there was no statistically significant improvement in proteinuria level with all participants remaining nephrotic (7.27 ± 7.30 g/d). GFR level marginally declined to 60 ± 38 ml/min with one patient progressing to ESKD. There were 2 serious infections, an infusion-related reaction and leucopenia attributed to rituximab.

**Conclusion:**

Rituximab was ineffective when administered to adult patients with treatment-resistant primary FSGS with a high suPAR and evidence of podocyte activation.

## Graphical abstract

**Figure:**
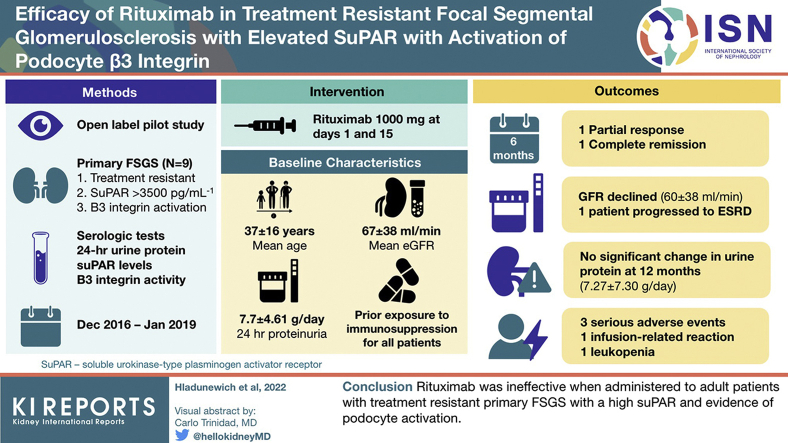

FSGS is described as a renal histologic lesion caused by diverse etiologies and pathologic processes, all of which can lead to podocyte injury and depletion.^1^ Patients with primary FSGS typically present with nephrotic syndrome, focal and segmental lesions on light microscopy, no definable immune complex deposition on immunofluorescence microscopy, and widespread foot process effacement on kidney biopsy electron microscopy examination. Spontaneous remissions are rare (<5%), and if patients are untreated and/or unresponsive to immunosuppression, the disease typically progresses to ESKD in 6 to 8 years in 50% of patients.^2^, ^3^, ^4^ Those with severe nephrotic syndrome (proteinuria level >10 g) have a worse prognosis and can expect to progress to ESKD in 3 to 5 years if unresponsive.^2^, ^3^, ^4^ Furthermore, the FSGS lesion recurs after transplantation in approximately a third of patients with severe primary disease contributing significantly to graft loss.^5^ Thus, primary FSGS poses a significant burden on patient health and well-being and health care resources.

In primary FSGS presenting with severe nephrotic syndrome, the presence of a circulating factor that results in podocyte effacement and disruption of the glomerular filtration barrier has been supported by several experimental and clinical studies.^10^, ^11^, ^12^, ^13^, ^6^, ^7^, ^8^, ^9^ Recent insights into podocyte biology have identified suPAR, a myeloid cell-derived circulating factor, which connects innate-immune function to the maintenance of the slit diaphragm through its ability to form signaling complexes with other transmembrane proteins, including activation of podocyte αvβ3 integrin.^14^ Activation of this receptor and its downstream pathways results in activation of small guanosine triphosphatases (e.g., Rac1), leading to podocyte foot process effacement, proteinuria, glomerular damage, and loss of renal function. In suPAR-transgenic mice, variable amounts of renal disease with proteinuria, loss of kidney function, and glomerulosclerosis, characteristics of FSGS, were noted.^15^ As such, both suPAR and evidence of activation of the podocyte β3 integrin have been proposed as a key mechanism for primary FSGS and as potential biomarkers.

Immunosuppressive treatments have been found to improve proteinuria and slow progression, but the side effects of current options that include high-dose prolonged corticosteroids, cytotoxic agents, and calcineurin inhibitors are significant, whereas efficacy is limited.^16^, ^17^, ^18^, ^19^ Rituximab is a genetically engineered, chimeric, murine/human monoclonal antibody directed against the CD20 antigen found on the surface of normal and malignant pre-B and mature B cells. Furthermore, rituximab may have a direct podocyte modulating effect by cross-reactivity with SMPDL-3b protein and regulation of acid sphingomyelinase essential for the lipid-raft compartmentalization of the podocyte plasma membrane and for the organization and signaling of podocytes in general.^20^ This potential direct effect on podocyte integrity, independent of its known effect on selective depletion of the B cell clone, supports rituximab as an attractive option to consider for the treatment of immunologically mediated FSGS. To date, only case reports and a small open-label trials exist to suggest that rituximab might prove effective in patients with FSGS, and these reports primarily included children and patients with postrenal transplant recurrence, wherein additional therapies were part of the treatment regimen.^21^, ^22^, ^23^, ^24^, ^25^, ^26^, ^27^

In this nonblinded, open-label pilot study, we tested the efficacy of rituximab in steroid-resistant or intolerant adults with primary FSGS. As part of the protocol, we also measured markers of disease activity and restricted trial entry to patients with both high levels of serum suPAR and evidence of activation of the podocyte β3 integrin. In addition to reporting on the trial results, we report on the sizable population of patients with FSGS who failed screening for inclusion on the basis of these biomarkers and a control population with other forms of glomerular disease. We hypothesized that rituximab may be an effective therapy in patients with FSGS and evidence of disease activation.

## Methods

### Study Subjects, Inclusion, and Exclusion Criteria

Participating patients were adults (>18 years of age) with primary FSGS. All biopsy reports were reviewed by the investigators to confirm evidence of diffuse foot process effacement (>80%) on electron microscopy (MAH, FCF, SMS). All study participants had proteinuria level ≥3.0 g/24 h and an estimated GFR ≥ 40 ml/min per 1.73 m^2^, using the 4-variable Modification of Diet in Renal Disease equation as published in the National Kidney Foundation—Chronic Kidney Disease guidelines.^28^ The rationale for the GFR criteria was that a patient with severely reduced GFR is more likely to have significant interstitial and glomerular scarring that would indicate irreversible injury. Finally, only patients with a suPAR level >3500 pg/ml with evidence of β3 integrin activation were included (microflow image >1). All subjects provided informed consent as per the Declaration of Helsinki for Medical Research Involving Human Subjects.

Other exclusions included the collapsing variant of FSGS, as it is rare and has been associated with an aggressive course, patients with medical conditions that may cause FSGS (e.g., HIV, lymphoma, heroin use), or those with a secondary form of FSGS that can be associated with hyperfiltration injury (e.g., massive obesity, vesicoureteral reflux, or renal mass reduction). Furthermore, patients with active infections, malignancy within the preceding 5 years, and type 1 or 2 diabetes mellitus were excluded. Women who were pregnant or nursing were also excluded for safety reasons.

### Definition of Treatment-Resistant FSGS

Treatment resistance or intolerance was defined as persistent or increasing proteinuria level (≥3.0 g), despite angiotensin-converting enzyme inhibitor/angiotensin-receptor blocker treatment as tolerated and a minimum of 8 weeks of prednisone therapy, a trial of calcineurin inhibitor for ≥3 months, cytotoxic therapy, and/or contraindication/intolerance to such therapy (e.g., osteoporosis/osteonecrosis). Patients exhibiting partial response to immunosuppressive treatment but remaining nephrotic required a minimal washout period before initiating rituximab to avoid overimmunosuppression and consequent risk of serious adverse events. Nevertheless, to avoid risk of worsening of their underlying FSGS disease, in those with some response, investigators were permitted to apply the following rules in regard to immunosuppressive therapies: cytotoxic therapy discontinued at least 6 months before initiation of rituximab; adrenocorticotropic hormone and/or mycophenolate mofetil discontinued at least 30 days before initiation of rituximab; calcineurin inhibitors tapered and discontinued within 60 days after the first rituximab dose, and prednisone reduced to ≤10 mg/d at least 30 days before receiving the rituximab infusion.

### Study Design and Protocol

This was a nonblinded, open-label study using rituximab provided by Genentech Pharmaceuticals. The study was performed at the Mayo Clinic and the University Health Network. Research ethics boards at both sites approved the study. Screening began in December of 2016 with enrollment during 2017 and 2018, and follow-up was completed by January 2019. The conduct and reporting of the study followed the Strengthening the Reporting of Observational Studies in Epidemiology guidelines for cohort studies (Supplementary Table S1). Before initiating active therapy, target blood pressure (BP) (systolic <140 mm Hg) was achieved during a 3-month run-in period. Angiotensin receptor blockers were used preferentially because they were better tolerated, with minimal cough or angioedema. The dose was increased at 2-week intervals until target BP was achieved or until intolerable side effects occurred. Additional medications were added as necessary in patients whose BP control was not at target at the discretion of the attending nephrologist. As part of the standard of care for patients with nephrotic syndrome and severe hyperlipidemia, patients were started on a statin increased to the maximum recommended or tolerated dose. Finally, all patients received dietary counseling to maintain a low salt diet (2–3 g/d) and a dietary protein target intake of 0.8 g/kg ideal body weight/d of protein throughout the duration of the study. Once stabilized, further escalations to drugs that block the renin-angiotensin system or the dose of lipid-lowering agents were not permitted. Dose reductions, however, were guided by side effects (i.e., hyperkalemia, hypotension, and myalgia).

Rituximab was infused i.v. on days 1 and 15 at a dose of 1000 mg. Established site infusion protocols were used, but all patients received preinfusion treatment with acetaminophen 1000 mg p.o. (give 30–60 minutes before rituximab), diphenhydramine (Benadryl) 50 mg oral (give 30–60 minutes before rituximab), and methylprednisolone 100 mg (SoluMedrol) in 0.9% sodium chloride to a total of 50 ml (200 ml/h completed 30 minutes before the start of rituximab infusion). After rituximab infusion, patients were started on double-strength Bactrim 3 times a week (or its equivalent) for pneumocystis pneumonia prophylaxis. This treatment continued until the B cells (CD19/CD20+) were replete (>15 cells/μl on peripheral blood flow cytometry).

At each visit, patients were questioned on their symptoms and possible side effects of therapy. Physical examination included the measurement of BP and body weight. Fasting blood samples and aliquots from 24-hour urine collections were taken at baseline and then at 1, 3, 6, and 12 months. Measurements included serum concentrations of creatinine, electrolytes, liver function tests, blood glucose, cholesterol profile (triglycerides, high-density lipoprotein cholesterol, low-density lipoprotein cholesterol), albumin, immunoglobulins (IgG, IgM, and IgA), and flow cytometry for CD19 and CD20. The GFR was estimated by the Modification of Diet in Renal Disease equation. Proteinuria was monitored by 24-hour urine collections with simultaneous urine creatinine measurements to ensure collection completeness. At each time point, blood was also sampled for suPAR and evidence of podocyte β3 integrin activation was obtained.

### Study Outcomes

The primary outcome measure was the change in proteinuria 12 months post-treatment. Complete remission was defined as proteinuria level <0.3 g/d; partial remission as reduction in proteinuria by >50% with a final urine protein level <3.0 g/d, but >0.3 g/d; incomplete remission as reduction in proteinuria level ≥50%, but residual proteinuria still >3.0 g/d and no response defined as worsening serum creatinine level >30% above baseline and/or <50% reduction in proteinuria or worsening of proteinuria. Secondary outcomes measured at 1, 3, 6, and 12 months post-therapy included changes from baseline suPAR level and activation of podocyte β3 integrin as indicated by relative AP5 activity, which is an antibody used to detect the active state of β3 integrin. Finally, changes in other measures of the nephrotic syndrome, including improvements in serum albumin and cholesterol profile and documented side effects and toxicity, were noted.

### Laboratory Determinations

Most values were determined by site-specific laboratory evaluations. Serum SuPAR concentration was determined by quantitative sandwich enzyme immunoassay technique (Quantikine Enzyme-Linked Immunosorbent Assay Human uPAR Immunoassay, R&D Systems and by Virgates) specified by manufacturer’s protocol. These samples were evaluated in a blinded manner at a central laboratory (JR). An additional 20 samples from patients with nephrotic syndrome secondary to other histologic causes than FSGS (e.g., membranous, membranoproliferative glomerulonephritis) served as an additional control group. The baseline sample was run in real time as it defined inclusion, and subsequent samples were batched and run at the end of the study. Activation of integrin in comparison with healthy controls is a measure that evaluates the podocyte-damaging effect of suPAR in the blood samples of patient with FSGS. To quantitatively evaluate the effect of patient sera with FSGS on podocyte β3 integrin activity, a conditionally immortalized human podocyte cell line was cultured at 37 °C for 14 days for complete differentiation. The cells were then incubated in 5% to 10% of patient serum with FSGS for 24 hours with recombinant suPAR protein as a positive control. Next, the cells were fixed with 4% paraformaldehyde and proceeded for immunofluorescence staining for AP5 (Blood Center of Wisconsin) and paxillin (Millipore). After immunostaining, confocal (Leica) images were taken to quantify the AP5 and paxillin intensity for each sample treatment. Paxillin signal is used to correct AP5 signal. The relative AP5 signal (AP5/paxillin ratio) from each patient serum is then normalized against that of normal blood donor included in each assay for final report. To control for suPAR specificity, the cells were co-incubated with both FSGS sera and suPAR-blocking antibody.

### Statistical Analysis

Descriptive statistics were calculated for all variables of interest. Continuous measures were summarized using mean ± SD, whereas categorical measures were summarized using counts and percentages. Paired testing was used to evaluate changes over time. The Fisher exact test was used to compare proportions. An analysis of variance was used to compare responses by dose with the Tukey test used for between-group comparisons. A *P* < 0.05 was deemed statistically significant. All analyses were carried out using STATA version 16 (StataCorp, College Station, TX).

## Results

### Cohort Screening and Inclusion

Of the 38 patients with FSGS screened for the study who met all clinical and histologic criteria, only 13 qualified based on suPAR level cutoff and cellular β3 integrin activation profile, of which 9 consented to participate. Nonconsenting patients were either concerned about potential side effects associated with rituximab or could not travel for study visits. Baseline characteristics of the patients who qualified for the study and those who did not are displayed in Table 1. Of the 25 excluded patients, 21 were excluded on the basis of a serum suPAR level, 2 were excluded on the basis of inadequate AP5 activation, and an additional 2 were excluded for not meeting either criterion. There were no significant clinical or other laboratory differences between included and excluded patients with all having parameters compatible with the nephrotic syndrome. Control samples included patients with other forms of kidney disease, including membranous nephropathy, IgA nephropathy, and other disease entities (*n* = 16). These patients were also not different by clinical or other laboratory parameters than the FSGS cohort with an average GFR of 73 ± 28 ml/min and an average proteinuria of 7.74 ± 3.84 g/d (Table 1).

**Table 1:** Baseline characteristics in screen-failed and eligible patients

Laboratory measurements	Screen-failed patients *n* = 25	Eligible patients *n* = 13	Control samples *n* = 16	*P* value
Urine protein (g/d)	6.36 ± 2.47	8.20 ± 4.66	7.74 ± 3.84	0.25
GFR (ml/min)	72 ± 25	68 ± 33	73 ± 28	0.89
Serum albumin (g/l)	31 ± 6	30 ± 7	31 ± 7	0.94
Total cholesterol	7.4 ± 2.4	8.8 ± 5.6	6.7 ± 2.2	0.29
LDL cholesterol	4.6 ± 1.7	4.5 ± 2.5	3.7 ± 1.5	0.43
suPAR (pg/ml)	2679 ± 1033	4306 ± 888^a^	3212 ± 857	<0.001
AP5 ratio	1.21 ± 0.33	1.59 ± 0.53	NA	0.005

GFR, glomerular filtration rate; LDL, low-density lipoprotein; NA, not applicable; SuPAR, soluble urokinase-type plasminogen activator receptor.

a *P* < 0.5 compared to both screen-failed patients and control samples. There was no significant difference between screen-failed patients and control samples.

### Baseline Characteristics of the Treated Cohort

The cohort had an average age of 37 ± 16 years. Approximately half of the cohort was male (56%) with most being White (67%). All, but 1 patient, were stable on either monotherapy (7 patients) or dual blockade (1 patient) of the renin-angiotensin system before the run-in phase with no noted changes in urine protein. The single patient not on renin-angiotensin system blockade did not tolerate the therapy owing to hypotension. Previous exposure to multiple immunosuppressive agents was noted in all patients. At the time of the baseline evaluation, immunosuppression regimens included high-dose prednisone monotherapy (1 patient), calcineurin monotherapy (1 patient), prednisone with calcineurin inhibitor (1 patient), mycophenolate mofetil with calcineurin inhibitor (2 patients), or triple therapy with prednisone, calcineurin inhibitor, and mycophenolate mofetil (4 patients). Other population baseline characteristics are displayed in Table 2. Mean 24-hour protein was 7.70 ± 4.61 g/d, mean serum albumin was 30 ± 7 g/dl, and baseline eGFR was 67 ± 38 ml/min. As per protocol, all baseline suPAR values were >3500 pg/ml with evidence of β3 integrin activation with average values of 4120 ± 1169 pg/ml and 1.56 ± 0.59 pg/ml, respectively.

**Table 2:** Treatment response

Patient characteristics	Baseline *n* = 9	1 mo *n* = 9	3 mo *n* = 9	6 mo *n* = 9	12 mo *n* = 8; 1 ESKD	*P* value
Urine protein (g/d)	7.70 ± 4.61	6.79 ± 5.09	7.16 ± 7.64	5.94 ± 4.61	7.27 ± 7.30	0.46
GFR (ml/min)	67 ± 38	57 ± 38	66 ± 37	63 ± 37	60 ± 38	0.02
Systolic BP (mm Hg)	127 ± 18	124 ± 22	123 ± 19	129 ± 22	128 ± 17	0.57
Diastolic BP (mm Hg)	83 ± 11	82 ± 11	81 ± 9	82 ± 10	86 ± 9	0.53
Serum albumin (g/l)	30 ± 7	29 ± 7	31 ± 7	33 ± 6	30 ± 7	0.27
Total cholesterol	8.4 ± 6.0	8.2 ± 3.4	7.2 ± 3.0	7.3 ± 3.5	7.0 ± 3.1	0.60
LDL cholesterol	3.8 ± 1.7	5.3 ± 3.0	4.1 ± 2.4	4.6 ± 3.2	4.6 ± 2.7	0.20
SuPAR (pg/ml) (R&D)	4120 ± 1169	3730 ± 1229	4231 ± 1871	4491 ± 2217	3788 ± 1836	0.41
SuPAR (pg/ml) (Virgates)	6507 ± 2284	7226 ± 3811	7759 ± 3811	7519 ± 4359	6415 ± 2320	0.32
AP5 ratio	1.56 ± 0.59	1.17 ± 0.17	1.13 ± 0.34	1.15 ± 0.30	1.24 ± 0.27	0.06

BP, blood pressure; ESKD, end-stage kidney disease; GFR, glomerular filtration rate; LDL, low-density lipoprotein; SuPAR, soluble urokinase-type plasminogen activator receptor.

### Treatment Response

**Figure 1:**
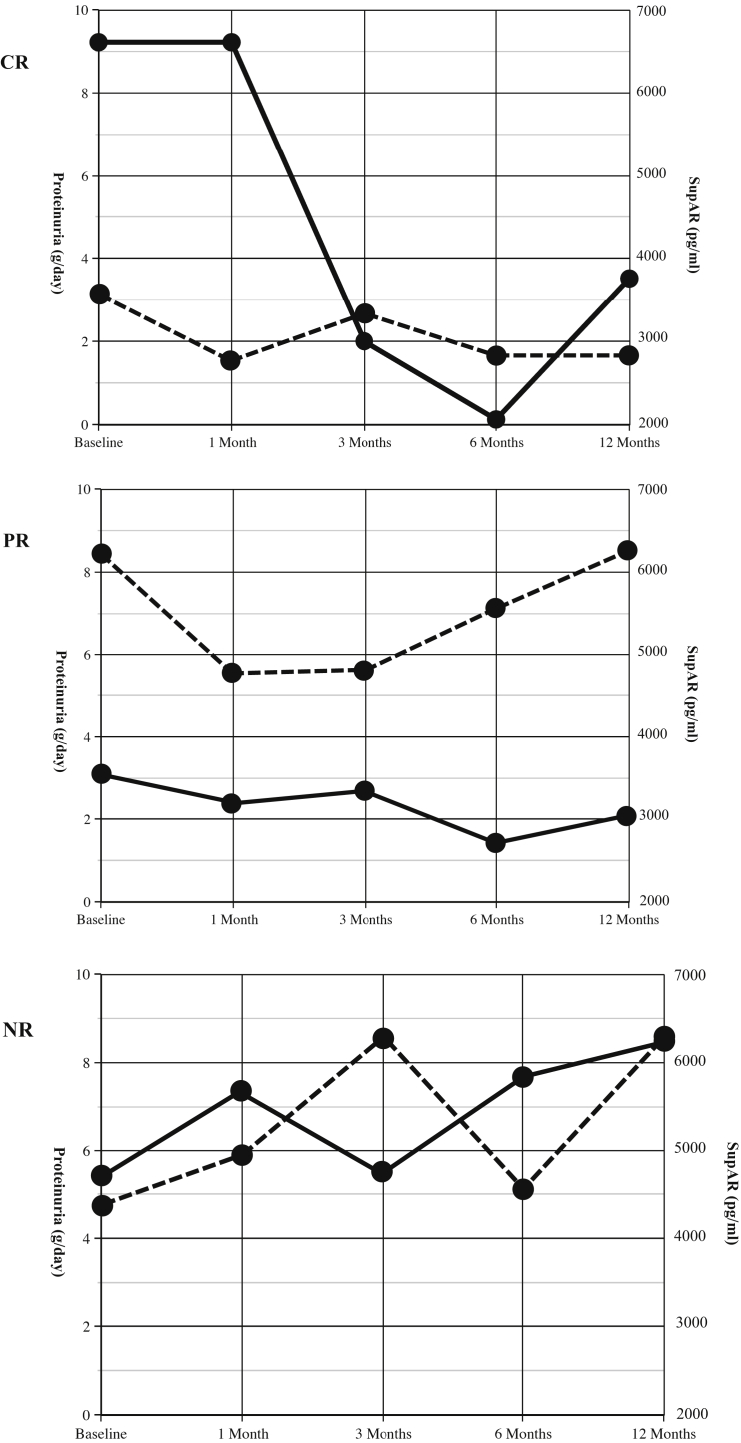
Proteinuria and corresponding serum SuPAR levels. Urine protein levels at baseline and 1, 3, 6, and 12 months with corresponding suPAR levels in 3 representative patients. Where panel CR is the patient with a complete remission at 6 months, PR is the patient with a partial remission at 6 months whereas NR demonstrates a patient with no response. Proteinuria is the solid line whereas suPAR is the dashed line. SuPAR, soluble urokinase-type plasminogen activator receptor.

There was no significant change in urine protein at 12 months compared with the baseline value (7.70 ± 4.61 vs. 7.27 ± 7.30 g) with no patients in remission at 12 months (Table 2). At 6 months, one patient had a partial response and one a complete remission (Figure 1). The single patient who responded had a normal GFR level. In the others, GFR levels declined significantly from 67 ± 38 to 60 ± 38 ml/min with 1 patient progressing to ESKD (*P* = 0.02). As such, no measure of proteinuria was available at the 12-month follow-up visit. Other measures of the nephrotic syndrome, including albumin and low-density lipoprotein cholesterol, were also not significantly different from baseline values at 12 months (Table 2). In the overall cohort, BP remained controlled throughout the trial. Despite the planned removal of other immunosuppressive agents, at the end of the trial, 2 patients remained on prednisone ≥5 mg/d, 3 remained on calcineurin inhibitors, and 3 remained on both as withdrawal of immunosuppression was deemed too precarious owing to lack of any response to rituximab. Rituximab therapy had no impact on the chosen biomarkers with no significant change in either suPAR as measured by R&D or Virgates or activation of β3 integrin (Table 2).

### Adverse Events

During the year of follow-up, there were 16 adverse events recorded, of which 3 were deemed as serious. One patient was hospitalized with flu-like symptoms and severe vomiting resulting in acute kidney injury who subsequently recovered, a second developed pneumonia who also recovered, and a third patient was hospitalized with a significant worsening in serum creatinine who progressed rapidly to ESKD in the context of persistent severe nephrotic syndrome. There were 2 adverse events deemed related to rituximab therapy including an infusion-related reaction and a decreased white blood cell level that recovered without sequelae. Other adverse events included nausea, vomiting, headaches, dizziness, weakness, cramps, headache, and a maculopapular rash, none of which were deemed by the investigator related to the rituximab therapy based on the timing of the events.

## Discussion

Given the nature of suPAR as an innate-immune circulating factor and the expanding use of rituximab in autoimmune diseases, this study was designed to evaluate the effectiveness of rituximab in adult patients with treatment-resistant primary FSGS, while incorporating information from plasma suPAR levels and the serum effects on podocyte β3 integrin activation. Of the 38 patients screened for the study who met the clinical criteria, only 13 qualified based on a serum suPAR level >3500 pg/ml and evidence of β3 integrin activation (microflow image >1). In the 9 patients with FSGS who consented to participate, rituximab was ineffective at producing a sustained remission. All patients at 12 months remained nephrotic with an overall significant decline in GFR level, including a single patient who progressed to ESKD. Only a single patient with a preserved GFR level responded completely, and another had a partial response by 6 months, but both relapsed by 12 months of follow-up (Figure 1). It is possible that these patients would have benefited from retreatment with rituximab at month 6, but this was not part of the protocol. Adverse events were significant with 3 classified as serious.

Success with the use of rituximab has been noted in the pediatric literature and after post-transplant FSGS recurrence albeit in the context of multitargeted immunosuppression and often plasmapheresis.^21^, ^22^, ^23^, ^24^, ^25^, ^26^, ^27^ The safety and efficacy of rituximab were evaluated in a multicenter series of 22 patients, aged 6 to 22 years, with severe steroid-dependent nephrotic syndrome or steroid-resistant, but cyclosporin-sensitive idiopathic nephrotic syndrome.^29^ Remission was induced in 3 of the 7 patients with proteinuria, and ≥1 immunosuppressive treatments could be withdrawn in 19 patients (85%), with no relapse of proteinuria. In another study, 54 children with idiopathic nephrotic syndrome dependent on either steroid or calcineurin inhibitor were randomized to receive either rituximab or ongoing standard immunosuppression.^30^ The experimental arm had a relapse rate of 18% compared with 48% in the control arm at 3 months and had a significantly higher probability of being free of long-term immunosuppression. Similarly, in a study of both children and adults with steroid-dependent or frequently relapsing nephrotic syndrome, including 8 patients with FSGS, rituximab decreased both the need for steroid maintenance and the number of relapses.^31^ These studies suggest that rituximab can be useful as a sparing or replacement immunosuppressive agent in FSGS noted to be responsive to other immunosuppressive therapies.

Nevertheless, even in steroid-resistant nephrotic syndrome, rituximab may result in remission in children. In a recent systematic review that included 226 children, response to rituximab was noted in 39.2% of children with steroid-resistant nephrotic syndrome secondary to FSGS.^32^ Studies that include only adult patients are more limited. A recent systematic review and meta-analysis included 16 observational studies that described the outcome after rituximab therapy in 51 adult patients with FSGS.^33^ The study noted a complete remission rate of 43% and a partial remission rate of 11%. The relapse rate of patients treated with rituximab was 47% in a mean follow-up period of 18.7 ± 9 months. Unlike our study, only a few of the included patients with FSGS were completely treatment resistant with most being steroid-dependent or frequently relapsing FSGS. Our study, in contrast, included treatment-resistant patients many on multitargeted immunosuppression. The lack of response to rituximab was further illustrated by our inability to safely wean off other immunosuppressive treatments in most of the patients. The single patient who responded had a preserved GFR and had only failed steroid monotherapy, suggesting in multidrug-resistant disease, rituximab does not seem to be an effective immunosuppressive therapy. Furthermore, 2 serious adverse events related to infections were noted.

In an effort to preselect the study patient group and to enrich the FSGS cohort with patients with high serum suPAR level and activation of podocyte β3 integrin, we used a serum level of suPAR >3500 pg/ml with evidence of β3 integrin activation (microflow image >1). SuPAR level has been previously found to be elevated in patients with FSGS with discriminatory power compared with other glomerular diseases.^10^ SuPAR may also reach very high levels in patients with recurrent disease post-transplantation.^10^ Furthermore, this 20 to 50 kilodalton circulating protein can be partially removed by plasmapheresis, and beneficial responses in proteinuria can be observed in cases where suPAR levels drop below a certain threshold where there is reduced activation of the podocyte β3 integrin.^34^ Our control population, which included patients with proteinuria level >3.0 g/24 h owing to other disease entities (e.g., IgA nephropathy, membranous nephropathy) had lower levels of suPAR than those with primary FSGS who met the inclusion criteria (3212 ± 857 vs. 4306 ± 888 pg/ml, respectively), but higher levels than those patients with FSGS who were screen failures (2679 ± 1033 pg/ml). This is similar to results noted using samples from the Neptune Cohort wherein significant overlap in the suPAR level was noted between patients with FSGS, minimal change disease, IgA nephropathy, and membranous nephropathy.^35^ In this mixed cohort, suPAR concentration at baseline inversely correlated with eGFR and the urine suPAR-to-creatinine ratio positively correlated with the urine protein-to-creatinine ratio.

Using serum suPAR in patients with nephrosis with biopsy-proven findings and otherwise multidrug-resistant FSGS did not prove to be a discriminatory biomarker, as noted by the high screen failure rate among patients equally nephrotic with biopsy-proven FSGS. This result is consistent with recent meta-analysis that noted suPAR alone could not distinguish patients presenting with FSGS from those in remission.^36^ β3 integrin activation assays can provide additional insights into patient stratification because integrin activation is the downstream event triggering cellular injury and may be different based on different suPAR isoforms or proteolytic fragments that measure similar suPAR amounts, yet with a different biological response. Moreover, the ability of suPAR to serve as a biomarker for FSGS may require additional co-factor analysis, such as genotype for APOL1^37^ or the presence of anti-CD40 autoantibodies.^38^ This is further supported by recent large studies suggesting suPAR to be a robust marker of innate-immune activation during inflammation,^39^^,^^40^ cardiovascular mortality,^41^ and chronic kidney disease incidence and progression.^41^ The enzyme-linked immunosorbent assay system measuring suPAR is important when attempting to use suPAR level as a biomarker for FSGS. Although both the R&D enzyme-linked immunosorbent assay and the Virogates enzyme-linked immunosorbent assay have been found to be useful,^42^ the latter had an improved ability to separate FSGS from healthy patients or from patients with other glomerular diseases.^42^ Although the cell-based integrin activation assay using podocytes exposed to patient serum has been used on a case-by-case basis, this study highlights difficulties to evaluate integrin activation in higher throughput fashion and on repeated sampling. Perhaps other cell lines with engineered β3 integrin expression such as transfected K562 cells^43^ may be used.

In summary, rituximab does not seem to be effective in adult patients with severe treatment-resistant FSGS with high serum suPAR level and podocyte integrin activation. Furthermore, in FSGS, when accompanied by severe nephrosis, rituximab may be hazardous. Whether rituximab in patients with FSGS with lower suPAR/integrin activity assessment would be useful requires study. Our study suggests that multidrug-resistant FSGS may be unique with respect to biomarkers and mechanistic pathways. Understanding the mechanisms in each patient through biomarker testing may ultimately guide more personalized treatment regimens that are more effective and avoid side effects.

## Disclosure

JR is a co-founder and co-chair of the scientific advisory board of Walden Biosciences. SSH is a member of the scientific advisory board of Walden Biosciences. All the other authors declared no competing interests.

## Supplementary Material

Supplementary File (PDF)

**Table S1.** Strengthening the Reporting of Observational Studies in Epidemiology (STROBE) Guidelines for Cohort Studies.

**STROBE Statement** (PDF)
